# Comparison of blood pressure values and expression of genes associated with hypertension in children before and after hematopoietic cell transplantation

**DOI:** 10.1038/s41598-021-88848-7

**Published:** 2021-04-29

**Authors:** Wojciech Strojny, Kinga Kwiecińska, Kamil Fijorek, Michał Korostyński, Marcin Piechota, Walentyna Balwierz, Szymon Skoczeń

**Affiliations:** 1 Department of Oncology and Hematology, University Children’s Hospital, Krakow, Poland; 2 Department of Oncology and Hematology, Institute of Pediatrics, Jagiellonian University Medical College, Wielicka 265, 30-662 Kraków, Poland; 3 Department of Statistics, Cracow University of Economics, Kraków, Poland; 4 Department of Molecular Neuropharmacology, Institute of Pharmacology PAS, Kraków, Poland

**Keywords:** Predictive markers, Paediatric cancer, Hypertension, Genetics research, Paediatric research, Risk factors

## Abstract

Hypertension is a well-known late effect of hematopoietic cell transplantation (HCT), but no markers predicting its development are known. Our aim was to assess short-term blood pressure (BP) values and expressions of hypertension-associated genes as possible markers of hypertension in children treated with HCT. We measured systolic blood pressure (SBP) and diastolic blood pressure (DBP), using both office procedure and ambulatory BP monitoring (ABPM) in children before HCT and after a median of 6 months after HCT. We compared the results with two control groups, one of healthy children and another of children with simple obesity. We also performed microarray analysis of hypertension-associated genes in patients treated with HCT and children with obesity. We found no significant differences in SBP and DBP in patients before and after HCT. We found significant differences in expressions of certain genes in patients treated with HCT compared with children with obesity. We concluded that BP values in short-term follow-up after HCT do not seem to be useful predictors of hypertension as a late effect of HCT. However, over expressions of certain hypertension-associated genes might be used as markers of hypertension as a late effect of HCT if this is confirmed in larger long-term studies.

## Introduction

Hypertension is a well-known late effect of hematopoietic cell transplantation (HCT). However, the precise mechanisms and time of its development in patients after HCT are not clear and there are no useful markers predicting its development^1–3^. It may be attributed to several factors, including the underlying condition, treatment, and secondary impact of other late effects of HCT, including obesity, which is also a well-known risk-factor of hypertension^4^. Currently the data are accumulating on genes associated with increased risk of various multigene diseases, including hypertension^5^. To our knowledge, no data are currently available on the expressions of these genes in pediatric patients treated with HCT.

Our purpose was to look for short-term blood pressure (BP) values and expressions of hypertension-associated genes as possible predictors of hypertension as a late effect of HCT.

We analyzed BP values in patients treated with HCT before, and in short-term (median 6 months) follow-up after the procedure. We used traditional office measurements and ambulatory BP monitoring (ABPM). We compared BP values in patients before and after HCT and in two control groups, one of healthy children and another of children with simple obesity. In a separate analysis we used microarrays to assess expressions of known hypertension-associated genes in patients before and after HCT and children with simple obesity.

## Results

### Office blood pressure measurements

Mean office SBP and DBP values were significantly higher in the obesity control group compared with the healthy control group (124/75.7 vs. 111/66.4 mmHg; P < 0.001), while no significant differences were found between the pre-HCT group or post-HCT group and the healthy control group (Table 1).

**Table 1:** Mean office SBP and DBP values in the pre-HCT group, post-HCT group and obesity control group, compared with the healthy control group.

Parameter (n of measurements)	Pre-HCT (70)	p	Post-HCT (54)	p	Obesity (75)	p	Healthy
Mean SBP, mmHg (SD)	108 (10.6)	0.227	104 (10.5)	0.021	124 (9.88)	< 0.001	111 (10.8)
Mean DBP, mmHg (SD)	66.9 (11.2)	0.841	62.8 (11.0)	0.196	75.7 (6.79)	< 0.001	66.4 (9.59)

Mean office SBP and DBP values were significantly higher in the obesity control group compared with both the pre-HCT group (124/75.7 vs. 108/66.9 mmHg; P < 0.001) and post-HCT group (124/75.7 vs. 104/62.8 mmHg; P < 0.001) (Table 2), while no significant differences were found between the pre-HCT and post-HCT groups (Table 3, results in paired tests see Supplementary Table S5).

**Table 2:** Mean office SBP and DBP values in the pre-HCT and post-HCT groups compared with the obesity control group.

Parameter (n of measurements)	Pre-HCT (93)	p	Post-HCT (77)	p	Obesity
Mean SBP, mmHg (SD)	108 (10.6)	< 0.001	104 (10.5)	< 0.001	124 (9.88)
Mean DBP, mmHg (SD)	66.9 (11.2)	< 0.001	62.8 (11.0)	< 0.001	75.7 (6.79)

**Table 3:** Mean office SBP and DBP values in the pre-HCT group compared with the post-HCT group.

Parameter*	Pre-HCT	Post-HCT	p
Mean SBP, mmHg (SD)	109 (11.2)	104 (10.5)	0.083
Mean DBP, mmHg (SD)	67.5 (12.0)	62.8 (11.0)	0.105

*n of measurements 56.

### Ambulatory blood pressure measurements

ABPM measurements revealed significantly higher values of mean arterial pressure (MAP) and MAP percentile, mean SBP, and DBP percentile in the obesity control group compared with the healthy control group. SBP percentile and mean DBP values were also higher in this group, but the differences were not significant. Comparisons of the pre-HCT group and the healthy control group revealed significant differences with respect to mean DBP values and MAP percentile. No significant differences were found between the post-HCT and healthy control groups (Table 4).

**Table 4:** ABPM parameters in the pre-HCT group the post-HCT group, and the obesity control group, compared with the healthy control group.

Parameter (n of measurements)	Pre- HCT (50)	p	Post-HCT (38)	p	Obesity (41)	p	Healthy
Mean SBP, mmHg (SD)	105 (15.4)	0.457	103 (10.6)	0.196	118 (9.66)	< 0.001	107 (7.86)
Mean SBP percentile (SD)	78.5 (10.4)	0.368	75.7 (3.50)	0.512	80.9 (8.95)	0.057	76.6 (4.73)
Mean DBP, mmHg (SD)	68.7 (12.2)	0.044	65.4 (9.42)	0.464	66.5 (6.8)	0.124	63.6 (4.6)
Mean DBP percentile (SD)	77.1 (7.86)	0.423	78.3 (6.71)	0.156	79.5 (7.70)	0.048	75.8 (3.44)
MAP, mmHg (SD)	80.6 (8.78)	0.598	79.4 (8.31)	0.925	84.1 (7.17)	0.023	79.6 (4.99)
MAP percentile (SD)	83.3 (11.1)	0.005	79.2 (8.70)	0.266	81.8 (8.53)	0.019	76.6 (4.73)

ABPM measurements revealed significantly higher SBP in the obesity control group compared with the pre-HCT group. Other ABPM parameters were also higher in the obesity control group, but the differences were not significant. Comparisons of the post-HCT group with the obesity control group revealed significantly higher mean SBP and SBP percentile in the obesity control group. Other ABPM parameters were also higher but the differences were not significant (Table 5). No significant differences in the ABPM parameters were found between the pre-HCT and post-HCT groups (Table 6; results in paired tests see Supplementary Table S6).

**Table 5:** ABPM parameters in the pre-HCT group and post-HCT group compared with the obesity control group.

Parameter (n of measurements)	Pre-HCT (53)	p	Post-HCT (41)	p	Obesity
Mean SBP, mmHg (SD)	105 (15.4)	< 0.001	103 (10.6)	< 0.001	118 (9.66)
Mean SBP percentile (SD)	78.5 (10.4)	0.381	75.7 (3.50)	0.018	80.9 (8.95)
Mean DBP, mmHg (SD)	68.7 (12.2)	0.401	65.4 (9.42)	0.694	66.5 (6.8)
Mean DBP percentile (SD)	77.1 (7.86)	0.264	78.3 (6.71)	0.588	79.5 (7.70)
MAP, mmHg (SD)	80.6 (8.78)	0.120	79.4 (8.31)	0.061	84.1 (7.17)
MAP percentile (SD)	83.3 (11.1)	0.595	79.2 (8.70)	0.331	81.8 (8.53)

**Table 6:** ABPM parameters in the pre-HCT group compared with the post-HCT group.

Parameter*	Pre-HCT	Post-HCT	p
Mean SBP, mmHg (SD)	107 (15.3)	105 (9.87)	0.442
Mean SBP percentile (SD)	75.4 (6.92)	75.9 (4.08)	0.585
Mean DBP, mmHg (SD)	66.2 (6.52)	66.9 (8.99)	0.714
Mean DBP percentile (SD)	77.4 (6.47)	78.1 (6.12)	0.766
MAP, mmHg (SD)	80.9 (6.63)	81.3 (6.78)	0.845
MAP percentile (SD)	82.9 (8.48)	79.2 (8.95)	0.277

*n of measurements 28.

### Microarray analysis

**Figure 1:**
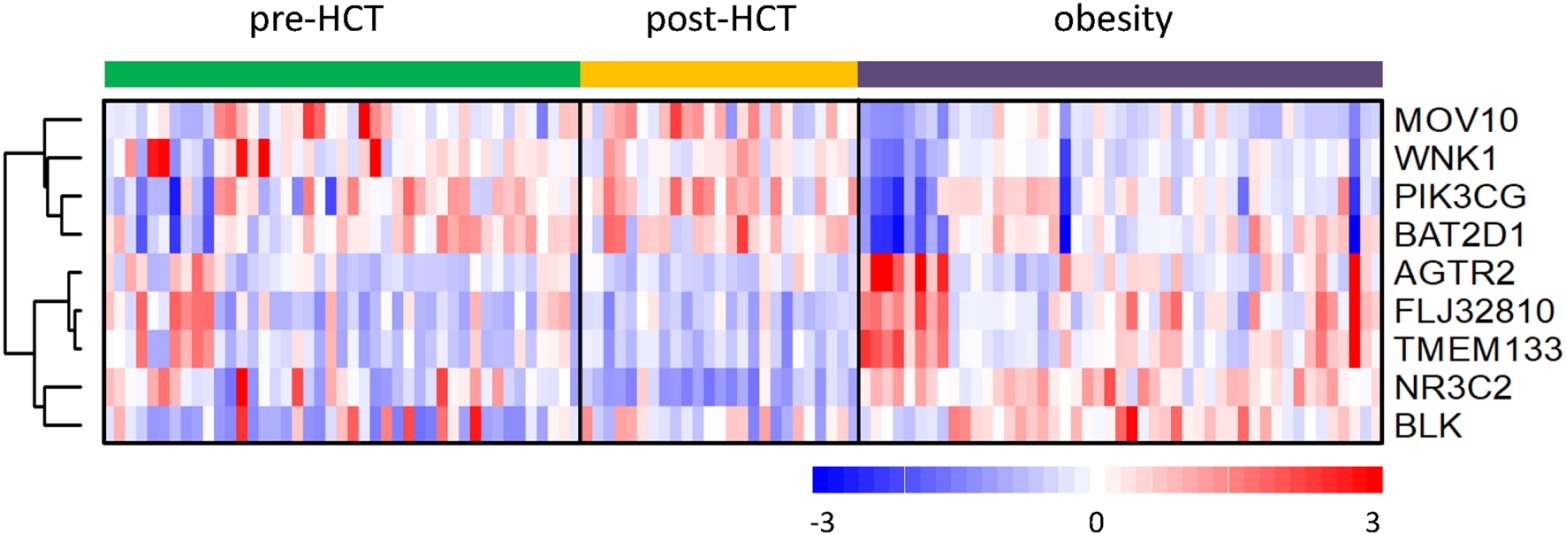
Differences in the expression of blood pressure-associated genes between pre-HCT, post-HCT, and obesity control group. Microarray results are shown as a heatmap and include the hypertension-associated genes for which significant differences in expressions between the study groups were found. Colored rectangles represent transcript abundance in the blood. The intensity of the shading is proportional to the standardized values (between − 3 and 3) from each microarray, as indicated on the bar below the heat map image. Hierarchical clustering was performed with the dChip (Ver 2/25/09+) software (https://sites.google.com/site/dchipsoft) using Euclidean distance and the average linkage method.

Microarray analysis revealed significant differences in expressions of genes associated with hypertension between the pre-HCT and obesity control group, as well as between the post-HCT group and the obesity control group. In the pre-HCT group we found significantly lower expression of AGTR2, BLK, FLJ32810 and TMEM133 genes, and significantly higher expression of MOV10 and WNK1 genes compared with the obesity control group. In the post-HCT group we found significantly lower expression of AGTR2, FLJ32810, NR3C2 and TMEM133 genes, and significantly higher expression of BAT2D1, MOV10, PIK3CG and WNK1 genes compared with the obesity control group. The MOV10 and WNK 1 genes were overexpressed in both pre-HCT and post-HCT group compared with the obesity control group. We found no significant differences in expressions of the hypertension-associated genes between the pre-HCT and post HCT groups, with only a few genes showing near-significant differences and no consistent pattern (including NR3C2 gene with higher expression in the pre-HCT group and PIK3CG with higher expression in the post-HCT group, both differences non-significant). Statistically significant differences in expressions of the respective genes are presented in Table 7. Gene expression profiles are presented in Fig. 1.

**Table 7:** Statistically significant differences of gene expressions between pre-HCT and post-HCT groups and the obesity control group.

Gene	Baseline mean	Experiment mean	t statistic	P value
**Pre-HCT vs. obesity**				
AGTR2	57.44	75.26	2.155	0.03457
BLK	177.06	222.69	2.935	0.004372
FLJ32810	97.74	120.44	2.326	0.022333
MOV10	475.49	398.28	− 2.353	0.021622
TMEM133	96.41	126.35	2.35	0.021231
WNK1	2294.1	1658.52	− 3.326	0.001469
**Post-HCT vs. obesity**				
AGTR2	52.43	75.26	2.749	0.007688
BAT2D1	2516.31	2123.82	− 2.359	0.021878
FLJ32810	84.94	120.44	3.888	0.000228
MOV10	497.86	398.28	− 3.045	0.004164
NR3C2	169.43	248.75	3.049	0.003794
PIK3CG	1390.81	1129.38	− 2.744	0.008415
TMEM133	83	126.35	3.509	0.000798
WNK1	2127.35	1658.52	− 3.08	0.003416

## Discussion

In our prospective analysis of patients treated with HCT compared with control groups of healthy children and obese children with no comorbidities we found no significant differences of BP values between the pre-HCT and the post-HCT groups, as well as the healthy controls. This was seen despite the fact that numerous patients have received drugs that may have contributed to hypertension, including glucocorticoids and ciclosporin. Also, the age, sex and disease status of these groups was not associated with a relative increase in the known risk of hypertension in any of these groups. We found elevated mean office BP values in the group of otherwise healthy obese individuals compared with other groups. This was consistent with a generally elevated prevalence of hypertension in this population, which may be over 30%, compared with over 10% in otherwise healthy children and adolescents without obesity^6–8^. BP parameters measured using ABPM generally followed a similar pattern with one exception, as mean DBP values and MAP percentile were significantly higher in the pre-HCT group than in healthy controls. Moreover, not all of the differences reached statistical significance.

HCT causes a variety of adverse effects, both early and late, that constitute significant health burden in patients treated with this therapeutic modality, affecting their quality of life and survival^9^. Late effects of HCT may appear over several years or decades after the procedure and the association between their emergence and HCT may not be evident^10^. Hypertension and obesity are very prevalent in contemporary societies^11–14^. Hypertension is also a well-known late effect of HCT^1–3^^.^ Hoffmeister et al. found that overall prevalence of hypertension in long-term survivors after pediatric HCT was 15%, which was up to three times higher than in the reference population^3^. The mechanisms of the emergence of hypertension in post-HCT patients are still not well understood. Contributing factors may include drugs used after the procedure, such as ciclosporin or glucocorticoids, as well as kidney or vascular injury caused by the procedure, however the relative importance of these factors has not been unequivocally proven and there are currently no known markers predicting the development of hypertension in individual patients^15–20^.

Our microarray analysis of the expressions of hypertension-associated genes revealed several significant differences in the gene expressions between the obesity control group, and the pre-HCT and post-HCT groups. A very interesting finding were significantly higher expressions of certain hypertension-associated genes in patients treated with HCT (both before and after the procedure) compared with obese individuals with no comorbidities. We found no significant differences in the expressions of the assessed genes between the pre-HCT and post-HCT groups.

The evidence of the role of genetic mechanisms in the pathogenesis of hypertension is accumulating^21–24^. This issue was comprehensively reviewed by Ehret and Caulfield, who listed 12 genes associated with monogenic types of hypertension and 43 genetic variants that are associated with multigene pathogenesis of hypertension^5^. In our study we compared expressions of these genes (and a few more new additions) in patients before and after HCT, as well as healthy and obese controls. Comparing patients before HCT with obese controls we found significant overexpression of four genes (AGTR2, BLK, FLJ32810 and TMEM133) in obese individuals, which could have been expected. However, quite unexpectedly, we found significant overexpression of two genes (MOV10 and WNK1) and near-significant overexpression of another gene (SH2B3) in patients before HCT. Similar findings were seen in the comparison of patients after HCT and obese controls, where significant overexpression of four genes (AGTR2, FLJ32810, NR3C2 and TMEM133; three of them overlapping the former comparison) was found in obese subjects, while significant overexpression of another four genes (BAT2D1, MOV10, PIK3CG and WNK1) was found in patients after HCT. The differences between patients before and after HCT were not significant and no consistent pattern was found. Of the genes overexpressed in patients before or after HCT, WNK1 is associated with a monogenic type of hypertension, while BAT2D1, MOV10, PIK3CG are genetic variants associated with BP^5,25^. MOV10 and WNK 1 genes were overexpressed both before and after HCT compared with the obesity control group. The altered expression of the genes was found in mononuclear cells. These were host cells in patients before HCT, and graft cells in patients after HCT. The differences in gene expression in host and graft cells are known and are subject of research focused on transplant rejection and tolerance^26–30^. The effects of microenvironment on gene expression are also implicated with respect to tumor angiogenesis^31^. This suggests that donor immune and hematopoietic system may influence host tissues and vice versa. This may also include the pathways taking part in regulation of BP. However, to our knowledge no data have been published to date on expression of the genes that may have a role in the development of late effects of cancer treatment. These findings are intriguing, as they might suggest the effect of some host factors that are beyond the direct effects of the graft. Hypothetically, either an underlying condition that was an indication for HCT, a prior treatment (particularly glucocorticoids), an HCT procedure itself, or a combination of these factors, might have some, yet unexplained, effects on expressions of the genes that could play a role in the future development of hypertension.

Our study has several limitations. These include low numbers of patients in the study groups, variable age of patients in the study and children in the control groups (caused by the inclusion of individual patients at the extremes of the pediatric age range), heterogenous population of healthy controls, heterogeneous indications for HCT in the study group, difference in the number of patients between pre-HCT and post-HCT groups caused by a high number of patients who died or were lost to follow up due to complications of their underlying diseases or HCT procedure, observational single-center design and relatively short follow-up. Another limitation is lack of comparison of gene expressions between patients before/after HCT and healthy controls. This was originally performed, but for technical reasons we found the results non-reportable, and thus we have not included this data set in our analysis.

In conclusion, our results show that in short-term follow-up BP values in pediatric patients treated with HCT were not significantly different before and after the procedure despite the use of agents potentially implicated in the development of hypertension. No significant differences were found compared with healthy controls, while the BP values were significantly lower than in the obese controls (except for two ABPM parameters). This suggests that short-term BP values after HCT may not be a useful marker of the development of hypertension as a late effect. In an additional analysis we found overexpressions of individual hypertension-associated genes in patients before and after HCT compared with obese controls. This is an intriguing finding, as it might suggest a role of some genetic factors in the development of hypertension in patients treated with HCT. This is a preliminary research and has numerous limitations reviewed above in the discussion, but in our opinion it merits further research to investigate the possible role of altered expression of hypertension-associated genes in the development of hypertension in this group of patients. If this is confirmed in larger studies, such genes might be used as markers predicting long-term risk of hypertension in patients treated with HCT.

## Methods

We prospectively compared four groups of children.

The group of patients assessed before HCT (pre-HCT group) included 44 patients (31 boys, 13 girls) aged 1.5–19 years (median 9.9 years), consecutively referred to the Stem Cell Transplantation Centre of the University Children’s Hospital in Krakow. Characteristics of the pre-HCT group are presented in Supplementary Table S1. Indications for HCT are presented in Supplementary Table S2, and types of transplantation procedures in Supplementary Table S3. Patients with malignancies were referred for HCT in complete remission (leukemia) or at least in a very good partial remission (solid tumors). HCT procedures were performed from June 2009 to August 2012. Before HCT all patients underwent a standard screening including detailed assessment of cardiac function (echocardiography) to exclude cardiomyopathy or other cardiovascular abnormalities. None of the patients had either cardiovascular disease, or a prior history or diagnosis of hypertension. None of the patients received antihypertensive treatment before and/or after HCT.

The group of patients assessed after HCT (post-HCT group) included 27 children (20 boys, 7 girls), aged 2.8–19.5 years (median 11.2 years) from the pre-HCT group in whom repeated investigations were performed after a median of 6 months after HCT. Characteristics of the post-HCT group are presented in Supplementary Table S4. The 6-month period of analysis was chosen because corticosteroids and immunosuppressive therapy are usually discontinued earlier than 6 months after HCT and therefore they would not affect BP results. The post-HCT group did not include 17 children from the pre-HCT group, of whom 12 were lost to follow up before reaching the time point of 6 months after HCT and 5 children died before they could be included in the post HCT group (the causes of death were complications of HCT or disease progression). Systemic glucocorticoids were used in 25 children in the post-HCT group to treat complications of HCT. Moreover, they received other immunosuppressive agents, including tacrolimus, mycophenolate mofetil, ciclosporin and etanercept. During the second follow up assessment five children still received tapered doses of immunosuppressive agents other than glucocorticoids (including ciclosporin).

Two control groups were recruited. The healthy control group included 26 children (11 boys, 15 girls, age range 4.3–16.0 years, median age 12.2 years). These were family donors, siblings of HCT patients or other healthy children unrelated to the patients. They had no acute or chronic diseases currently or in the past and had laboratory test results (complete blood count, and serum alanine aminotransferase and creatinine levels), as well as body mass index values (BMI: mean 20.5 kg/m^2^, SD 4.42; BMI percentile: mean 67.3, SD 27.6; BMI SDS: mean 0.71, SD 0.84) within normal ranges. The obesity control group consisted of 49 children with simple obesity (22 boys and 27 girls; age range 3.4–17.8 years, median 13.5 years). In this group we included children with simple obesity (BMI: mean 31.7 kg/m^2^, SD 5.77; BMI percentile: mean 99.6, SD 0.83; BMI SDS: mean 3.28, SD 0.97) who had no other acute or chronic disease currently or in the past and were consecutively referred to an obesity clinic at our center. We excluded children with monogenic obesity, endocrine disorders, or other systemic diseases. Obesity diagnosis was based on anthropometric measurements performed at the Department of Children’s and Adolescent Endocrinology, Institute of Pediatrics, Jagiellonian University Medical College. Permanent Ethical Committee for Clinical Studies of the Medical College of the Jagiellonian University approved the study protocol (KBET/96/B/2008); study registration number: NN 407 198737. All experiments were performed in accordance with relevant guidelines and regulations. All parents/guardians, adolescent and adult patients, as well as subjects included in control groups, signed a written informed consent before blood sample collection. An informed consent for publication was obtained from all patients and subjects included in control groups or their parents/guardians, where applicable.

### Office blood pressure measurements

Included systolic BP (SBP) and diastolic BP (DBP) and were performed at 1–2 min intervals. An oscillometric BP monitor was used (Omron Healthcare Europe, Hoofdorp, the Netherlands), fitted with cuffs appropriate to the child’s age. Recorded BP values were averages of the last two measurements. BP values were compared to centile charts according to the fourth report from the National High Blood Pressure Education Program (NHBPEP)^32^.

### Ambulatory blood pressure monitoring

ABPM methodology was described in detail in our previous study^33^. 24-h ABPM were performed using the same type of monitor (Spacelab, Snoqualmie, WA, USA) that was fitted at all times by the same expert nurse. All children regardless of age were able to tolerate the procedure^8^. The monitor recorded BP and pulse rates in 20-min intervals at daytime and in 30-min intervals at night. Mean SBP and DBP values and mean arterial pressure (MAP) were calculated for 24 h, daytime, and nighttime. A licensed ABPM software was used. As some of the patients were < 120 cm of height and < 5 years of age and normative values for ABPM for these groups are lacking, we only compared relative ABPM values between respective patient groups and no comparisons with normative values were performed^8,34,35^.

### Microarray analysis

Methodology was described in detail in our previous study^33^. At the time of obtaining the samples the patients have not received glucocorticoids. Blood samples were collected from the pre-HCT, post-HCT and obesity control groups, mononuclear cells were separated, total RNA extraction was performed, and microarray analysis was performed using the GeneChip Human Gene 1.0 ST Array (Affymetrix, Santa Clara, CA, USA). Initial processing of the microarray data was performed using GeneChip Operating Software. DTT data were then transferred using the Transfer Tool software (Affymetrix, Santa Clara, CA, USA). Chip quality was assessed according to the guidelines by Affymetrix. Raw data were processed using the model-based expression index implemented in dChip (Cheng Li Lab, Ver 2/25/09+). After background subtraction, the data were normalized using quintile normalization. The signal was taken as the measure of mRNA abundance derived from the level of gene expression. Characteristics of the studied genes related to hypertension on the basis of Ehret and Caulfield^5^ and EntrezGene database are presented in Supplementary Table S5.

### Statistical analysis

All the continuous variables are presented as mean and standard deviation (SD) values. The categorical variables are presented as frequencies and percentages. The Shapiro–Wilk test was used to test for the normality of the data. To examine differences between the independent groups (normally distributed data) Student’s t-test or ANOVA (Analysis of Variance) were used. The Mann–Whitney/Kruskal–Wallis tests for non-normally distributed variables were used. The power of the microarray study was estimated using the R pwr package (version 1.2–0). Cohen’s d effect size (1.01) was calculated using average within-group SD 0.99 (for log2 values), assuming two-fold as expected change of fold. Significance levels of differences between the experimental groups were calculated for each microarray probe set using a t-test. P-values below 0.05 were considered statistically significant. The statistical analyses were carried out using R 3.0 software (Bioconductor).

## Supplementary Information

Supplementary Information

## Data Availability

Data generated or analyzed during this study are included in this published article (and its Supplementary Information files). The datasets generated for this study can be found in the GEO Series accession number GSE88852 (http://www.ncbi.nlm.nih.gov/geo/query/acc.cgi?acc=GSE88852).
