# Supplementary material for: Comparison of blood pressure values and expression of genes associated with hypertension in children before and after hematopoietic cell transplantation
Source: Sci Rep. 2021 Apr 29;11:9303. doi: 10.1038/s41598-021-88848-7 (PMC8085120; doi:10.1038/s41598-021-88848-7)
Supplement: Supplementary file 1 — Supplementary Information [file 41598_2021_88848_MOESM1_ESM.docx]

**Supplementary material to:**

**Comparison of blood pressure values and expression of genes associated with hypertensionn children before and after hematopoietic cell transplantation**

**Wojciech Strojny^1^, Kinga Kwiecińska^2^, Kamil Fijorek^3^, Michał Korostyński^4^, Marcin Piechota^4^, Walentyna Balwierz^2^, Szymon Skoczeń^2*^**

^1^ Department of Oncology and Hematology, University Children’s Hospital, Krakow, Poland

^2^ Department of Oncology and Hematology, Institute of Pediatrics, Jagiellonian University Medical College, Krakow, Poland

^3^Department of Statistics, Cracow University of Economics, Krakow, Poland

^4^Department of Molecular Neuropharmacology, Institute of Pharmacology PAS, Krakow, Poland

Corresponding Author

Szymon Skoczeń, Department of Oncology and Hematology, Institute of Pediatrics, Jagiellonian University Medical College, Wielicka 265, 30-662 Kraków, Poland, [szymon.skoczen@uj.edu.pl](mailto:szymon.skoczen@uj.edu.pl)

Supplementary Table S1. Characteristics of the pre-HCT group

| Patients, n | 44 |
| --- | --- |
| Sex, n | boys 31, girls 13 |
| Age (years), range (median) | 1.5 - 19 (9.9) |
| Chemotherapy before HCT, n (%) | 31 (71%) |
| BMI (kg/m^2^), mean (SD) | 19.0 (4,21) |
| BMI percentile, mean (SD) | 61.3 (31.1) |
| BMI SDS, mean (SD) | 0,52 (1.33) |
| Chemotherapy line before HCT, n  1  2  ≥ 2 | 18  10  3 |
| Local radiotherapy, n  Cranial, n (dose)  Testes, n (dose)  Involved fields in Hodgkin Lymphoma, n | 10  6 (12Gy-5, 18Gy-1)  2 (12Gy/24Gy, 18Gy/18Gy)  2 |
| Time since diagnosis and patient selection, (years), range (median) | 0.08 –12.9 (1.2) |
| Conditioning regimen based on busulfan, n (%) | 24 (55%) |
| Total body irradiation – 12Gy/6 fractions, n (%) | 11 (25%) |
| GvHD prophylaxis, n (%)  CSA  MTX+CSA | 4 (9%)  34 (77%) |
| GvHD, n (%) | 25 (57%) |

BMI – body mass index HCT – hematopoietic cell transplantation, GvHD – graft-versus-host disease, CSA- ciclosporin, MTX – methotrexate, SD- standard deviation

Supplementary Table S2. Indications for HCT in the pre-HCT group.

| Diagnosis | n ( %) |
| --- | --- |
| Neoplastic diseases – total | 32 (73) |
| Acute lymphoblastic leukemia | 17 (38.6) |
| Acute myeloblastic leukemia | 5 (11.3) |
| Acute bilineage leukemia | 1 (2.3) |
| Chronic myelocytic leukemia | 1 (2.3) |
| Hodgkin lymphoma | 2 (4.6) |
| Myelodysplastic syndrome | 1 (2.3) |
| Juvenile myelomonocytic leukemia and acute myeloblastic leukemia | 1 (2.3) |
| Neuroblastoma | 4 (9) |
| Non-neoplastic diseases – total | 12 (27) |
| Severe aplastic anemia | 4 (9) |
| Diamond-Blackfan anemia | 1 (2.3) |
| Fanconi anemia | 1 (2.3) |
| Chronic granulomatous disease | 3 (6.8) |
| Autoimmune lymphoproliferative syndrome | 1 (2.3) |
| Hyper IgM syndrome | 1 (2.3) |
| Congenital neutropenia | 1 (2.3) |

HCT – hematopoietic cell transplantation,

Supplementary Table S3. Types of HCT procedures

| Type of HCT, n(%) | | Disease, n |
| --- | --- | --- |
| Allogeneic,  n=38 (86%) | MUD, 25 (57) | Acute lymphopblastic leukemia, 12  Acute myeloblastic leukemia, 5  Acute bilineage leukemia, 1  Chronic myelocytic leukemia, 1  Severe aplastic anemia, 1  Diamond-Blackfan anemia, 1  Fanconi anemia,1  Chronic granulomatous disease, 2  Congenital neutropenia,1 |
|  | MSD, 9 (20) | Acute lymphoblastic leukemia, 3  Severe aplastic anemia, 2  Juvenile myelomonocytic leukemia and acute myeloblastic leukemia, 1  Chronic granulomatous disease, 1  Hyper IgM syndrome, 1  Myelodysplastic syndrome, 1 |
|  | MFD, 4 (9) | Acute lymphoblastic leukemia, 2  Severe aplastic anemia, 1  Autoimmune lymphoproliferative syndrome, 1 |
| Autologous,  n= 6 (14%) | | Hodgkin lymphoma, 2  Neuroblastoma, 4 |

HCT – hematopoietic cell transplantation, MFD – matched family donor; MSD – matched sibling donor; MUD – matched unrelated donor

Supplementary Table S4. Characteristics of the post-HCT group

| Patients, n | 27 |
| --- | --- |
| Sex, n | boys 20, girls 7 |
| Age (years), range (median) | 2.8 – 19.5 (11.2) |
| BMI (kg/m^2^), mean (SD) | 18.3 (3.47) |
| BMI percentile, mean (SD) | 53.0 (35.4) |
| BMI SDS, mean (SD) | 0.37 (1.26) |
| Time from HCT to the second assessment (months), range (median) | 5.9-19.1 (6.3) |
| Systemic glucocorticoids, n (%) | 25 (93%) |
| Oral budesonide treatment, n (%) | 7 (26%) |
| Cumulative dose of glucocorticoids (equivalent of prednisone, mg/m^2^), range (median) | 29 – 9758 (1531) |
| Time of systemic glucocorticoid therapy (days), range (median) | 1 – 315 (73) |
| Time since discontinuation of glucocorticoids (months), range (median) | 0 – 14.3 (3.5) |
| Time from discontinuation of immunosuppressive treatment to the second assessment* (months), range (median) | 0 – 9.5 (2) |

* 22 patients were included

HCT – hematopoietic cell transplantation, SD – standard deviation

Supplementary Table S5. Mean office SBP and DBP values in the pre-HCT group compared with the post-HCT group using paired tests (27 patients).

| Parameter* | Pre-HCT | Post-HCT | p |
| --- | --- | --- | --- |
| Mean SBP, mmHg (SD) | 109 (11.2) | 104 (10.5) | 0.083 |
| Mean DBP, mmHg (SD) | 67.5 (12.0) | 62.8 (11.0) | 0.105 |

Supplementary Table S6. ABPM parameters in the pre-HCT group compared with the post-HCT group using paired tests (27 patients).

| Parameter* | Pre-HCT | Post-HCT | p |
| --- | --- | --- | --- |
| Mean SBP, mmHg (SD) | 107 (15.3) | 105 (9.87) | 0.442 |
| Mean SBP percentile (SD) | 75.0 [75.0;75.0] | 75.0 [75.0;75.0] | 1.000 |
| Mean DBP, mmHg (SD) | 66.2 (6.52) | 66.9 (8.99) | 0.714 |
| Mean DBP percentile (SD) | 75.0 [75.0;75.0] | 75.0 [75.0;75.0] | 1.000 |
| MAP, mmHg (SD) | 80.9 (6.63) | 81.3 (6.78) | 0.845 |
| MAP percentile (SD) | 80.0 [75.0;90.0] | 79.2 [75.0;75.0] | 0.277 |

Supplementary Table S7. Characteristics of the studied genes

| probe set | gene | Accession | EntrezGene |
| --- | --- | --- | --- |
| 7902367 | ACADM | NM_000016 | acyl-Coenzyme A dehydrogenase nuclear gene encoding mitochondrial protein |
| 8171449 | ACE2 | NM_021804 | angiotensin I converting enzyme (peptidyl-dipeptidase A) 2, mRNA. |
| 7936028 | ACTR1A | NM_005736 | ARP1 actin-related protein 1 homolog A, mRNA. |
| 8054077 | ACTR1B | NM_005735 | ARP1 actin-related protein 1 homolog B, mRNA. |
| 7930627 | ADRB1 | NM_000684 | adrenergic, beta-1-, receptor, mRNA. |
| 7924987 | AGT | NM_000029 | angiotensinogen (serpin peptidase inhibitor, mRNA. |
| 8083240 | AGTR1 | NM_031850 | angiotensin II receptor, type 1, transcript variant 4, mRNA. |
| 8169492 | AGTR2 | NM_000686 | angiotensin II receptor, type 2 , mRNA. |
| 7897745 | AGTRAP | NM_020350 | angiotensin II receptor-associated protein, transcript variant 1, mRNA. |
| 7958784 | ALDH2 | NM_000690 | aldehyde dehydrogenase 2 family (mitochondrial), nuclear gene encoding mitochondrial protein, mRNA. |
| 7965359 | ATP2B1 | NM_001001323 | ATPase, Ca++ transporting, plasma membrane 1, transcript variant 1, mRNA. |
| 7907310 | BAT2D1 | NM_015172 | BAT2 domain containing 1, mRNA. |
| 8125017 | BAT5 | NM_021160 | HLA-B associated transcript 5, mRNA. |
| 8144625 | BLK | NM_001715 | B lymphoid tyrosine kinase, mRNA. |
| 7927723 | C10orf107 | BC041932 | chromosome 10 open reading frame 107, mRNA |
| 8104758 | C5orf23 | BC022250 | chromosome 5 open reading frame 23, mRNA |
| 7926506 | CACNB2 | NM_201596 | calcium channel, voltage-dependent, beta 2 subunit, transcript variant 2, mRNA. |
| 7904036 | CAPZA1 | NM_006135 | capping protein (actin filament) muscle Z-line, alpha 1, mRNA. |
| 7912316 | CASZ1 | NM_001079843 | Homo sapiens castor zinc finger 1, transcript variant 1, mRNA. |
| 8059393 | CUL3 | NM_003590 | Homo sapiens cullin 3, mRNA. |
| 8153363 | CYP11B1 | NM_000497 | Homo sapiens cytochrome P450, family 11, subfamily B, polypeptide 1, nuclear gene encoding mitochondrial protein, transcript variant 1, mRNA. |
| 8153373 | CYP11B2 | NM_000498 | cytochrome P450, family 11, subfamily B, polypeptide 2, nuclear gene encoding mitochondrial protein, mRNA. |
| 7936050 | CYP17A1 | NM_000102 | cytochrome P450, family 17, subfamily A, polypeptide 1, mRNA. |
| 7990391 | CYP1A1 | NM_000499 | cytochrome P450, family 1, subfamily A, polypeptide 1, mRNA. |
| 8096875 | ENPEP | NM_001977 | Homo sapiens glutamyl aminopeptidase (aminopeptidase A) mRNA. |
| 8096050 | FGF5 | NM_004464 | Homo sapiens fibroblast growth factor 5, transcript variant 1, mRNA. |
| 7963328 | FIGNL2 | NM_001013690 | fidgetin-like 2 (FIGNL2), mRNA. |
| 7943349 | FLJ32810 | ENST00000298815 | cDNA FLJ32810 fis, clone TESTI2002729, weakly similar to oligophrenin 1 gene:ENSG00000165895 |
| 7950492 | FLJ33790 | NM_001039548 | hypothetical protein FLJ33790, mRNA. |
| 8144643 | GATA4 | NM_002052 | GATA binding protein 4, mRNA. |
| 8063668 | GNAS | NM_000516 | GNAS complex locus, transcript variant 1, mRNA. |
| 8007904 | GOSR2 | NM_004287 | golgi SNAP receptor complex member 2, transcript variant A, mRNA. |
| 8056327 | GRB14 | NM_004490 | growth factor receptor-bound protein 14, mRNA. |
| 8097957 | GUCY1A3 | NM_000856 | guanylate cyclase 1, soluble, alpha 3 , mRNA. |
| 7904717 | HFE2 | NM_213653 | hemochromatosis type 2 (juvenile), transcript variant a, mRNA. |
| 7996563 | HSD11B2 | NM_000196 | hydroxysteroid (11-beta) dehydrogenase 2, mRNA. |
| 7945146 | KCNJ5 | NM_000890 | potassium inwardly-rectifying channel, subfamily J, member 5, mRNA. |
| 7949172 | MAP4K2 | NM_004579 | mitogen-activated protein kinase kinase kinase kinase 2, mRNA. |
| 8123176 | MAS1 | NM_002377 | MAS1 oncogene, mRNA. |
| 8083494 | MME | NM_007288 | membrane metallo-endopeptidase, transcript variant 2a, mRNA. |
| 7911767 | MMEL1 | NM_033467 | membrane metallo-endopeptidase-like 1, mRNA. |
| 7904050 | MOV10 | NM_020963 | Moloney leukemia virus 10, mRNA. |
| 7912496 | MTHFR | NM_005957 | 5,10-methylenetetrahydrofolate reductase (NADPH), mRNA. |
| 8026214 | NANOS3 | NM_001098622 | nanos homolog 3, mRNA. |
| 8104746 | NPR3 | NM_000908 | natriuretic peptide receptor C/guanylate cyclase C (atrionatriuretic peptide receptor C) , mRNA. |
| 8103094 | NR3C2 | NM_000901 | nuclear receptor subfamily 3, group C, member 2 , mRNA. |
| 7941537 | PACS1 | NM_018026 | phosphofurin acidic cluster sorting protein 1 |
| 8135363 | PIK3CG | NM_002649 | phosphoinositide-3-kinase, catalytic, gamma polypeptide |
| 7929388 | PLCE1 | NM_016341 | phospholipase C, epsilon 1 |
| 7946781 | PLEKHA7 | NM_175058 | pleckstrin homology domain containing, family A member 7 |
| 7994074 | SCNN1B | NM_000336 | sodium channel, nonvoltage-gated 1, beta (Liddle syndrome) |
| 7994058 | SCNN1G | NM_001039 | sodium channel, nonvoltage-gated 1, gamma |
| 7958749 | SH2B3 | NM_005475 | SH2B adaptor protein 3 |
| 8101992 | SLC39A8 | NM_022154 | solute carrier family 39 (zinc transporter), member 8 |
| 8085914 | SLC4A7 | NM_003615 | solute carrier family 4, sodium bicarbonate cotransporter, member 7 |
| 8013135 | SREBF1 | NM_001005291 | Homo sapiens sterol regulatory element binding transcription factor 1 (SREBF1), transcript variant 1, mRNA. |
| 7918569 | ST7L | NM_017744 | suppression of tumorigenicity 7 like , transcript variant 1 |
| 7966668 | TBX5 | NM_181486 | T-box 5 (TBX5), transcript variant 4 |
| 7943369 | TMEM133 | NM_032021 | transmembrane protein 133 |
| 7990400 | ULK3 | NM_001099436 | unc-51-like kinase 3 |
| 8086352 | ULK4 | NM_017886 | unc-51-like kinase 4 |
| 7999936 | UMOD | NM_003361 | uromodulin (uromucoid, Tamm-Horsfall glycoprotein), transcript variant 1 |
| 7952953 | WNK1 | NM_018979 | WNK lysine deficient protein kinase 1 |
| 8007363 | WNK4 | NM_032387 | WNK lysine deficient protein kinase 4 |
| 8016546 | ZNF652 | NM_014897 | zinc finger protein 652 |
